# The complete mitochondrial genome of *Xiphister atropurpureus* (Perciformes: Stichaeidae)

**DOI:** 10.1080/23802359.2017.1303349

**Published:** 2017-03-21

**Authors:** Hartnell College Genomics Group:, Laura Ayala, Joseph Becerra, Ga Hun Boo, David Calderon, Angelo DiMarco, Armando Garcia, Reuben Gonzales, Jeffery R. Hughey, Gustavo A. Jimenez, Thomas A. Jimenez, Jose Lopez, Natalia I. Marquez, Ana I. Meza, Pedro Ortega, Roberta K. Overman, Conrado Pena, Michael Porras, Danielle E. Rodriguez, Juan Solorio, Arnulfo Soria, Gabriel Suarez, Diana A. Tamayo, Frances L. Wong

**Affiliations:** a Division of Mathematics, Science, and Engineering, Hartnell College, Salinas, CA, USA;; b University Herbarium, University of California, Berkeley, Berkeley, CA, USA

**Keywords:** Mitogenome, Stichaeidae, xiphister, Xiphisterinae, zoarcales

## Abstract

Analysis of the marine black prickleback *Xiphister atropurpureus* Kittlitz using 76 bp paired-end Illumina sequences resulted in the assembly of its complete mitogenome. The mitogenome is 16,518 bp in length and contains an origin of light strand replication (OL), control region, 22 tRNA, 2 rRNA, and 13 protein-coding genes. Content and organization of the *X. atropurpureus* mitogenome is consistent with other teleost. Phylogenetic analysis of *X. atropurpureus* resolves it in a clade with another member of the Stichaeidae, *Chirolophis japonicus* Herzenstein.

The Stichaeidae fish family consists of entirely marine pricklebacks classified to six subfamilies, 35 genera, and 70 species (Eschmeyer & Fong 2017; Eschmeyer et al. 2017). Two mitogenomes have been deciphered for the family, these are *Chirolophis japonicus* subfamily Chirolophinae (Yang et al. 2016) and *Leptoclinus maculatus* Fries subfamily Lumpeninae (Swanburg et al. 2016). Here, we describe the mitogenome of *X. atropurpureus*, a member of the subfamily Xiphisterinae and common intertidal to subtidal species distributed from Kodiak Island, Alaska to Baja California, Mexico (Eschmeyer et al. 1983).

DNA was extracted from *X. atropurpureus* (Specimen Voucher- Hartnell College #262) collected from under a boulder in the mid intertidal at Pacific Grove, California (36°37′43.2**″**N, -121°55′17.5**″**W) following the protocol of Lindstrom et al. (2011). The 76 bp paired-end library construction and sequencing was performed by myGenomics, LLC (Alpharetta, GA) yielding 16,427,262 reads. The mitogenome was assembled by mapping the reads against the reference sequence *Chirolophis japonicus* (GenBank NC_028022) using the Medium-Low Sensitivity/Fast setting in Geneious 8.0 (Biomatters Limited, Auckland, New Zealand). The genes were annotated using MITOS (Bernt et al. 2013) and adjusted manually using NCBI ORF-finder (https://www.ncbi.nlm.nih.gov/orffinder/). Alignment of the mitogenome to other perciforms was accomplished with MAFFT (Katoh & Standley 2013). The maximum**-**likelihood analysis was performed using complete mitogenome sequences with RaxML (Stamatakis 2014) in Galaxy (Giardine et al. 2005; Blankenberg et al. 2010; Goecks et al. 2010) using the GTR + gamma model and 1000 fast bootstraps. The tree was visualized with TreeDyn 198.3 at Phylogeny.fr (Dereeper et al. 2008).

The mitogenome of *X. atropurpureus* (GenBank KY657279) is 16,518 bp in length, with a base composition of 25.4% A, 27.6% T, 18.7% G, and 28.3% C. It is highly conserved in organization, as is typical of animals (Boore 1999). The mitogenome contains 22 tRNA (trnL and trnS are duplicated), 2 rRNA (rrnL, rrnS), and 13 genes involved in electron transport and oxidative phosphorylation. All 13 genes start with the ATG initiation codon except *cox1*, which initiates with GTG. Most of the 13 genes terminate with the TAA stop codon, however, *nd3* and *cob* terminate with TAG, and *cox2* and *nad4* with AGA. The *nd6* gene and eight tRNAs encode on the light-strand, with the remaining genes encoding on the heavy strand. The OL is located between trnN and trnC, and is 38 bp in length, and the control region is 854 bp. Phylogenetic analysis of *X. atropurpureus* resolves it with two Stichaeids in a clade with *C. japonicas*, paraphyletic with respect to *L. maculatus* (Figure 1).

**Figure 1.**
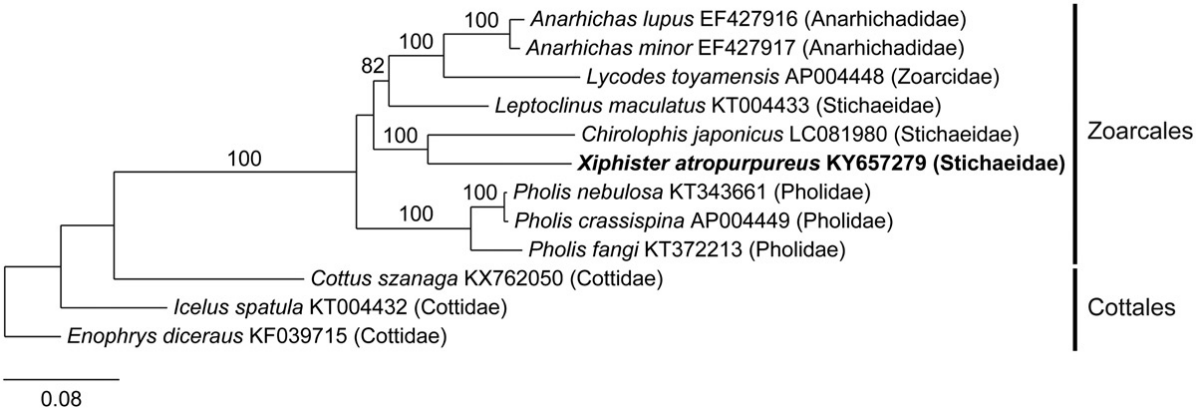
Maximum-likelihood phylogram of *Xiphister atropurpureus* and related teleost mitogenomes. Numbers along branches are RaxML bootstrap supports based on 1000 nreps (<75% support not shown). The legend below represents the scale for nucleotide substitutions.
